# Social ageing: exploring the drivers of late-life changes in social behaviour in mammals

**DOI:** 10.1098/rsbl.2021.0643

**Published:** 2022-03-02

**Authors:** Erin R. Siracusa, James P. Higham, Noah Snyder-Mackler, Lauren J. N. Brent

**Affiliations:** ^1^ School of Psychology, Centre for Research in Animal Behaviour, University of Exeter, Exeter, UK; ^2^ Department of Anthropology, New York University, New York, NY, USA; ^3^ Center for Evolution and Medicine, Arizona State University, Tempe, AZ, USA; ^4^ School of Life Sciences, Arizona State University, Tempe, AZ, USA; ^5^ School for Human Evolution and Social Change, Arizona State University, Tempe, AZ, USA

**Keywords:** ageing, evolution, mammals, social behaviour, senescence

## Abstract

Social interactions help group-living organisms cope with socio-environmental challenges and are central to survival and reproductive success. Recent research has shown that social behaviour and relationships can change across the lifespan, a phenomenon referred to as ‘social ageing’. Given the importance of social integration for health and well-being, age-dependent changes in social behaviour can modulate how fitness changes with age and may be an important source of unexplained variation in individual patterns of senescence. However, integrating social behaviour into ageing research requires a deeper understanding of the causes and consequences of age-based changes in social behaviour. Here, we provide an overview of the drivers of late-life changes in sociality. We suggest that explanations for social ageing can be categorized into three groups: changes in sociality that (a) occur as a result of senescence; (b) result from adaptations to ameliorate the negative effects of senescence; and/or (c) result from positive effects of age and demographic changes. Quantifying the relative contribution of these processes to late-life changes in sociality will allow us to move towards a more holistic understanding of how and why these patterns emerge and will provide important insights into the potential for social ageing to delay or accelerate other patterns of senescence.

## 1. Introduction

The formation of social connections within groups is a key mechanism that organisms use to cope with socio-environmental challenges ranging from finding food, to caring for offspring, to evading predators [1]. As a result, social interactions often play a key role in determining the resources that an individual has available to allocate to reproduction and maintenance. Studies across mammals have shown that the most socially integrated individuals have better health outcomes, increased survival and improved reproductive performance [2,3], making it clear that social relationships are a critical component of the fitness of many group-living species. It is increasingly appreciated that these social relationships are not static but are likely to change in form and function as individuals age. Recent work has demonstrated that humans and nonhuman animals show diverse and complex patterns of age-based changes in social behaviour [4,5]. For example, humans tend to show higher levels of social selectivity in older age, emphasizing emotionally meaningful relationships and reporting smaller social networks as a result [6,7]. Similar patterns have been observed in primates [8–12], whales [13], rodents [14,15] and deer [16], whereby older individuals interact with fewer social partners and spend less time on affiliative behaviour. While reduced social behaviour and social network size are clearly common in old age, it is also evident that ageing individuals often maintain interest in their social world and motivation to engage in social interactions [9,17]. In other cases, researchers have found that individuals do not appear to exhibit age-based differences in sociality [18,19] or have found that older individuals show increased affiliation [20,21].

It is therefore apparent that sociality, as with many traits, has the potential to show substantial variation in how it is expressed across the adult lifespan [4,22] and may have important downstream effects on other aspects of behaviour, physiology and life history. Yet while much attention has been given to understanding how sociality affects ageing patterns in animals [23–27], comparatively little work has looked at how senescence or other patterns of ageing affect late-life changes in social behaviour. There are well-established age-based changes in physiology, cognition, experience, and life history that might contribute to social ageing [28,29]. For instance, age-related declines in physical [30] and cognitive traits [31] are likely to affect social skills and may cause gradual declines in social behaviour with age as the result of senescence. By contrast, social behaviour may exhibit positive changes with age, which could be attributed to processes such as increasing skill or experience [32,33] or responses to changes in the force of natural selection with age [34]. Senescent declines might also lead organisms to adjust their social behaviour to compensate for lost resources or physiological deterioration with increasing age [12]. In this paper, we argue that by quantifying the relative contribution of these different processes to late-life changes in sociality, we can move towards a more holistic understanding of how and why patterns of social ageing emerge and gain a deeper understanding of their consequences for senescence and life-history evolution. These processes are not mutually exclusive and may operate simultaneously or synergistically to bring about late-life changes in social behaviour. This makes it all the more important to clearly identify cases where predicted changes in social behaviour with age overlap and to identify routes to disentangle the processes underlying those changes whenever possible. We aim to highlight this issue and to offer some strategies to resolve it.

**Figure 1.**
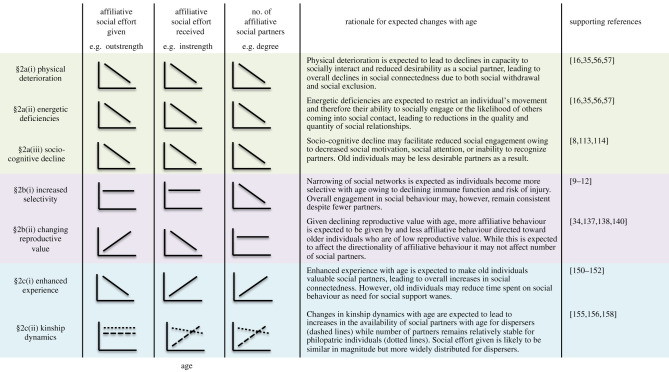
Challenges to disentangling explanations for age-related changes in adult social behaviour. Here, we make example predictions for how sociality might be expected to change with age according to each of the seven explanations outlined in the text, using a hypothetical mammal species that is group-living, with low levels of fission–fusion, where one sex is philopatric and the other disperses, and where both sexes experience declines in reproductive value with age. Explanations resulting from senescent decline have green shading, explanations that are secondary adaptations to senescence have purple shading, and explanations resulting from the positive effects of age and demographic changes have blue shading. In the predictions under ‘kinship dynamics’, dotted lines represent philopatric individuals, while dashed lines represent dispersers. We have chosen three measures of prosocial behaviour that have commonly been measured in the social ageing literature (e.g. [10,14,16,35]), although there are likely to be many additional metrics of interest, including centrality, clustering and betweenness, among others. We have included supporting references that either provide some empirical evidence that a given explanation might be driving social ageing and/or offer theoretical support for the predictions shown. We acknowledge that the predictions outlined here may change depending on the study system in question, requiring a clear understanding of an organism's ecology and life history. Regardless, our example predictions clearly show that it is likely to be challenging to quantify the relative contribution of these explanations by studying behavioural outcomes alone because predictions are similar for many explanations. This, in turn, indicates that integrative approaches involving longitudinal data, physiological markers and/or experiments are needed (see box 1).

To achieve these aims, we organize the potential drivers of social ageing into three types: (a) those that result from senescence across body systems; (b) those that result from adaptations to ameliorate the negative effects of senescence; and (c) those that result from positive effects of age and demographic changes. We demonstrate when and how predictions derived from these explanations overlap and make the case that phenotypic patterns will not be sufficient to tease overlapping explanations apart (see figure 1, below). We offer suggestions for systems and approaches where this issue might best be resolved (see box 1, below) and conclude by outlining the major priorities and challenges for future research.

Box 1. **Different approaches for studying social ageing in mammalian systems.**

Studies of ageing in mammals can be broadly placed into four categories, each with their own strengths in terms of the questions that can be asked and insights that can be revealed. **Experiments** allow researchers to isolate mechanisms underlying social ageing, and to sometimes document their consequences. For example, genetically modified rodent models, where features purported to influence social behaviour with age, such as metabolism, cognitive ability and sensory perception, are already established [36,37], provide the opportunity for laboratory-based experimental manipulation of social ageing [29]. Experiments can also uncover the causal direction of the relationship between sociality and ageing—whether features that change with age affect social behaviour, but also whether sociality can slow the pace of ageing.

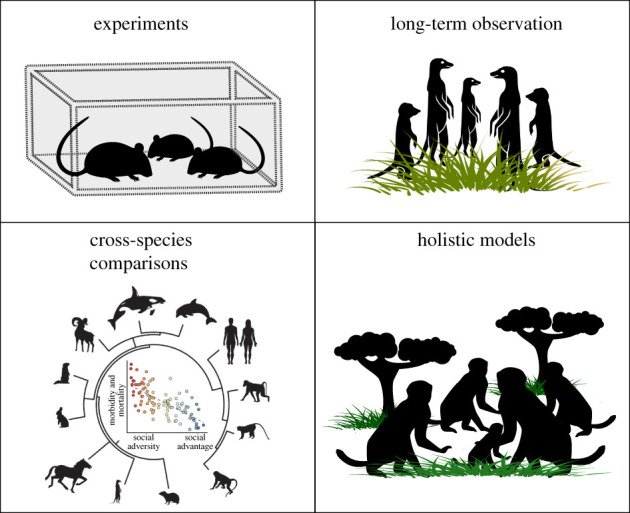

Studies where **long-term observations** of known individuals are possible permit not only cross-sectional but also within-subject study designs, allowing the separation of within-individual age-based changes in sociality from apparent age-related declines that are actually due to differences among cohorts or processes like selective disappearance [38,39]. Established long-term studies of wild or free-roaming rodents, mustelids, carnivores, ungulates, cetaceans and primates [38,40,41] allow social behaviour and evolution to be investigated under a natural range of socio-ecological conditions, provide valuable opportunities to document intra-individual consistency (or plasticity) of behaviours in response to ageing, and help to reveal the reasons why not all individuals in a population age in the same way [38].

Using social behaviour to cope with the challenges of group-living may have common evolutionary origins [1,2]. The features that affect behaviour across the lifespan can be **compared and contrasted across these species** to reveal universals of social ageing, similarities due to phylogenetic proximity, and the selective pressures driving social ageing processes. Species with phenotypes that are rare or at the extreme ends of a continuum are important for revealing how sociality and ageing intersect. For example, humans and some species of toothed whale are the only vertebrates known to exhibit a prolonged period of post-reproductive life, a trait believed to be associated with changes in the costs and benefits of social and reproductive behaviour as a female's relatedness to her group changes with age [42]. There are also social species in which social behaviour has no apparent association with age, such as the giraffe [43] and grey kangaroo [44], and these may be useful models in which to explore the reasons why social ageing may not always occur.

Animals that might be considered **holistic models** exhibit age-based changes in many of the features likely to influence social behaviour and have the capacity to support a wide range of study designs. For example, some of the best studied nonhuman primates, such as the rhesus macaque, are already established ageing models [45] known to exhibit age-related changes in physiology, cognition and immune function [46] but on a timescale compacted into a 3–4× shorter lifespan compared with humans. These features, combined with the existence of populations that are wild [10], free-roaming [47] and captive [48], make the rhesus macaque well suited as a holistic study system. (Cross-species comparison figure reproduced from [2, p. 1]. Images: TheNounProject/LELSAA/CC-By(Meerkat); TheNounProject/NickNovel/CC-By(Bighorn Sheep); TheNounProject/#7027/CC-By(Orca); TheNounProject/Matthews.Hall/CC-By(Bottlenose Dolphin); other icons by Nathalie Cary and Alice Kitterman/*Science*).

We focus explicitly on social ageing in group-living mammals. The current epidemic of social isolation among the elderly [49] has led to increasing interest in understanding the evolutionary basis of age-based changes in human social behaviour [9,12]. Mammals, in particular, offer potential for productive discourse between bio-gerontologists and evolutionary ecologists owing to their phylogenetic proximity to humans. Similar to humans, many mammal species live in stable groups with consistent pairwise interactions among group members [50] and show clear evidence of a link between social integration, health and longevity [2]. Furthermore, the changes that come with age, and which can influence behaviour, are shared in humans and many other group-living mammals. Studying how social behaviours change in response to these features in nonhuman mammals therefore has potential to offer insights into the physical, ecological and evolutionary drivers of social ageing in humans.

We define social ageing as changes in individual sociality throughout the adult life stage. This includes changes in an individual's social behaviour with age, changes in how others behave towards an individual as that individual getst older and changes in the emergent or latent phenotypes (e.g. social position or status) that arise as the result of these processes or other non-social behavioural changes such as altered space use. Given increasing interest in how social behaviour changes in old age [9,12,14], we only discuss changes that occur in adulthood, instead of across development from infancy to adulthood. Parental and reproductive behaviours are active areas of age-based research [51], but here we are primarily interested in the cooperative and competitive interactions in which group-living animals engage. However, we do discuss how changes in reproductive value and effort with age can moderate non-sociosexual behaviours. While apparent changes in sociality with age can emerge as a result of population-level processes such as selective disappearance [38,52], in this manuscript we are primarily interested in the processes that can lead to within-individual changes in sociality. We discuss the challenges and importance of differentiating these reasons for social ageing as being able to measure within-individual changes in social behaviour (and the associated fitness consequences) is particularly crucial for understanding how and when social ageing might evolve. Our aim is to provide a resource that will help guide future empirical work by encouraging researchers to quantify the relative contribution of these different explanations and in doing so facilitate a clearer understanding of how sociality and ageing intersect.

## 2. Explanations for social ageing in mammalian systems

### (a) Explanations for social ageing resulting from senescence across bodily systems

Here, we briefly summarize the evidence demonstrating how mammalian body systems tend to senesce, with examples of how this might impact social behaviour. We include the following: (i) physical deterioration of musculoskeletal and sensory systems; (ii) energetic deficiencies; and (iii) declines in sociocognitive architecture. Social ageing is a relatively new area of research [4] and clear empirical evidence of a link between bodily senescence and changes in social behaviour within adults as they age is not often available. We have instead endeavoured to provide plausible examples of what might occur (and could be investigated) when empirical evidence is lacking.

#### (i) Physical deterioration of musculoskeletal and sensory systems with age

Many mammals face losses in muscle mass (sarcopaenia), bone mass (osteoporosis) and muscle strength and functionality as they age [30,46,53–55]. Given that musculoskeletal health is central to mobility, dexterity and the ability to actively participate in social life, these declines are likely to affect social behaviour in myriad ways. For instance, in group-living mammals where individuals travel, forage or hunt as collective units, poor motor function may limit activity [8,35,56,57] and inhibit the ability of older individuals to keep up with units on the move. This may be particularly true in species where unit membership is highly fluid (i.e. with high levels of ‘fission–fusion’ sociality), such as many primates, cetaceans and ungulates [58,59]. Reduced fine motor control with age [46,60] may also limit engagement in affiliative behaviours important to the formation and maintenance of social relationships, such as grooming. Changes in body mass (reviewed in [61]) as well as tissue damage that accumulates with age, such as loss or breakage of teeth, tusks or antlers and tears to muscles and ligaments [46,62], can also hinder an individual's ability to compete or cooperate. In despotic social systems, this may open up opportunities to contest rank, leading to changes in social status [63,64] that could alter other group members' perception of an individual's value as a social partner. Age-related decline in tooth function [62,65,66] might also limit where animals can feed or graze, or increase the amount of time spent feeding to compensate for reduced efficiency, and thus impose restrictions on socality [67]. Habitat choice might be further restricted by the fact that older animals tend to have less brown adipose tissue [68,69] and thinner coats [70,71], reducing their ability to maintain homeothermy and meaning they are less likely to occupy exposed habitats, leading to increased isolation if these are areas frequented by the group. While physical deterioration imposes its own limitations on sociality, in many cases declines in physical features such as body mass, tooth function or fat stores are also likely to be tightly linked to energetic deficiencies with age and might facilitate changes in social behaviour in other ways. We discuss this further in §2a(ii) below.

The loss of sensory capabilities is also an important change that is expected to represent an acute challenge to the maintenance of social interactions and relationships with age. Recognition of, and communication with, others is essential to being able to take advantage of opportunities to cooperate, while avoiding competitors and costly conflicts [72]. Sensory systems, including hearing [46,73,74], vision [46,75] and olfaction [76–78] ,show consistent declines with age across mammals. Inability to recognize conspecifics or assess social status [79–81] may lead individuals to actively withdraw from interactions, and sensory deficiencies may affect the ability of individuals to effectively engage in affiliative behaviours [82], which could reduce the desirability of individuals as partners and lead to increased social isolation. Declines in sensory modalities may not only affect the ability of aged individuals to respond to, but also produce, social information. Older adults can also differ from younger conspecifics in the characteristics of the auditory, olfactory and visual cues they produce [73,83], potentially altering their ability to communicate and therefore interact.

#### (ii) Energetic deficiencies with age

As individuals age, they experience changes in energy regulation [84]. Food intake and absorption of nutrients can decline in older mammals [46,54], one potential cause of which is tooth wear with age [85], leading to reduced body fat and muscle mass, decreased bone mass, micronutrient deficiencies, immune dysfunction, and reduced wound healing [86,87]. The resulting declines in energy expenditure and activity levels [88] are likely to lead to changes in the amount or valence of social interactions with age. For instance, reduced energy levels may limit how far older individuals range and thereby reduce social connections [56,57,59]. Additionally, energetic deficiencies may either directly or indirectly, for example through associated declines in body mass [61], restrict engagement in prolonged energetically expensive interactions (e.g. physical contests), or lead individuals to change the types of social behaviours they use by switching from physical aggression to less energetically costly behaviours [56,89]. By hindering the ability to compete, energetic declines may facilitate opportunities for lower- ranking animals to contest rank and lead to changes in social status, similar to physical declines [63,64]. Energetic declines might also make old individuals less valuable as social partners because they are unable to spend as much time on important group behaviours like grooming [11], vigilance [90], and social foraging [91], leading to social exclusion. Finally, decreased metabolic heat production [69], which can operate in addition to physical changes such as senescent alopecia, is likely to be an important cause of declines in the ability to appropriately thermoregulate with age [92,93]. These changes in thermoregulatory ability may lead to social isolation as animals have to make behavioural decisions that prioritize temperature regulation over social interaction (i.e. adaptive allocation of resources, see §2b(i) below) or alternatively might lead old individuals to rely on alternative methods such as social thermoregulation [94], which might enhance connectedness [95].

#### (iii) Declines in sociocognitive architecture with age

Declines in general cognitive abilities [31,74,96], as well as memory [97] and motivation [29,98], are key aspects of biological senescence that may prevent individuals from being able to recognize social partners, effectively monitor their social environments or recall past interactions [79,81,99], thus limiting engagement in the social world with age. However, it has also been demonstrated that individuals might maintain interest in social relationships at the expense of the non-social world in the face of limited cognitive resources, leading to a preferential interest in social stimuli with age [9], although this is debated [100]. Changes in social interest and motivation can also occur independently of general cognitive losses and might result from age-related declines in regions of the brain strongly linked to social processes, and lead to reductions in social attentiveness, competence or social decision-making [101–103]. For instance, the prefrontal cortex and hippocampus are associated with the regulation of social behaviour in primates and other mammals [104,105] and exhibit strong age-related declines [74,106]. Gaze-following (or ‘social attention’) allows individuals to orient their attention to the same stimulus in the environment as conspecifics [107] and is an important skill for detecting socially relevant information and processing social cues. Social attention has been suggested to decline with increasing age in mammals [108,109] (although this varies across species [17]) owing to age-related deterioration of regions of the brain associated with gaze-processing [110]. Declines in the hippocampus can also affect information-processing abilities [105,111] and thereby the ability of individuals to attend and respond to social information and adjust their behaviour accordingly [112–114]. This might lead to increased social withdrawal, particularly from unfamiliar individuals with whom interactions are unpredictable [8,114].

The neuroendocrine system also plays a key role in the regulation and synchronization of social interactions [115] and shows substantial impairment with age, meaning that behaviour, along with other complex physiological processes including energy homeostasis, physical performance and cognition, is commonly disrupted [116]. These consequences for physiological processes can have their own downstream effects on social behaviour (as detailed in §§2a(i–iii), but neuroendocrine disruption with age might also directly affect sociality. For instance, age-based reductions of reward pathways linked to social behaviour [117,118] (e.g. the dopaminergic system) might translate to age-related changes in affiliative or agonistic interactions as the value of social interactions declines with age [118]. Age-related changes in the neuropeptide oxytocin, which plays a critical role in social bonding in many mammals, are likely to have important effects on social cognition and prosocial behaviour [119]. Dysregulation of the hypothalamic–pituitary–gonadal axis can lead to changes in testosterone levels with age and facilitate changes in social status [120]. Additionally, increased cortisol production with age as the result of senescence of the hypothalamic–pituitary–adrenal axis [121] might increase affiliative behaviour with age [122,123], although the relationship between glucocorticoids and sociality is complex and highly dependent on context and environment [124]. Elevated levels of glucocorticoids are also known to be associated with hippocampal ageing [125], which might have negative downstream effects on social behaviour [105].

### (b) Explanations for social ageing resulting from adaptations to ameliorate the negative effects of senescence

Cooperative and competitive interactions are a key means by which social organisms cope with challenges in their environment, from caring for offspring to accessing resources [1]. Yet the physical, cognitive and life-history changes that organisms face as they age might limit the capacity for, or shift the costs and benefits of, social interaction. Here, we discuss how individuals might show advantageous adjustments in social behaviour with age in response to these senescent declines. Specifically, we discuss how social ageing might result from: (i) increased selectivity and allocation of limited resources and (ii) changes in reproductive value and effort across the lifespan. Given that empirical evidence linking these explanations to social ageing is limited, we use theory to inform expected changes in social behaviour with age where available.

#### (i) Increased selectivity and allocation of limited resources with age

As individuals age, they should be expected to adjust the allocation of their time and energy to meet their changing needs and limited physical, sensory and cognitive capabilities in later life (see §2a). One of the constraints that older individuals are therefore likely to face is reduced time and energy or cognitive capacity for social interactions. Such limitations may promote adaptive narrowing of networks and select for increasing focus on important or preferred social partners [8,9,11,12]. In addition to physical and cognitive declines promoting increased selectivity in the choice of social partners, age-related reductions in immune function and healing ability (i.e. immunosenescence) [126–129] might similarly affect how individuals adjust their sociality with age. Given that social interactions play a central role in disease transmission [130], individuals might narrow their social networks and reduce social engagement with increasing age to avoid infection. This type of social avoidance is a common form of defence against pathogen transmission across species [131], and in some species, declines in parasite burdens with age might be an indication of these behavioural changes in older animals (e.g. [132]). Immunosenescence may also select for reduced risk-taking behaviour and competition avoidance. Older individuals might use milder forms of aggression [56,89], reduce their rate of engagement in agonistic behaviours [12,133], or withdraw from social interactions altogether to help minimize the chances of negative social interactions [56,133]. This might also explain increased attentiveness towards negative or threatening social stimuli with age (e.g. [134]). Finally, declines in food acquisition or nutrient processing with age may facilitate advantageous declines in social behaviour. For instance, acute hunger or depletion of energetic resources may reduce an individual's willingness to cooperate because of a shift towards self-preservation in the face of limited energy stores [135].

#### (ii) Changing reproductive value and effort across the lifespan

For many mammals, reproductive value (the relative contribution of an individual of a given age to the future of the population [136]) is a feature that declines with increasing age beyond the onset of reproduction and can interact with kinship dynamics (see §2c(ii) below) to play a crucial role in the way that individuals allocate their social effort with age [34,137]. Helping behaviour (or cooperation) is most likely to evolve between donors of low reproductive value and recipients of high reproductive value, given similar levels of relatedness between donors and potential receivers [34,137,138]. This is because individuals can maximize their indirect fitness benefits by helping relatives of high reproductive value and can maximize their direct fitness benefits by selfishly avoiding cooperative or altruistic behaviour when their own reproductive value is high. This fitness trade-off will shape not only when in life individuals should allocate time and energy to social behaviour but also who they should direct that social effort toward [34,137,138]. For instance, given that reproductive value declines with increasing adult age (and must eventually fall to zero as individuals approach maximum longevity), older individuals are expected to give more affiliative behaviour to younger relatives with higher reproductive value and receive less affiliation [139]. This might also lead to changes in social status as older individuals give up status to younger female relatives, as is seen in baboons (*Papio cynocephalus* [140]), macaques (*Macaca mulatta*; [141]) and langurs (*Semnopithecus entellus*; [142]). However, recent work has also theoretically demonstrated that an individual's reproductive effort (allocation to any current act of reproduction), rather than their reproductive value alone, is important for the evolution of social behaviour [34]. For example, mutual helping is less likely to evolve if individuals are reproductively active and therefore competing for breeding resources than if they are reproductively inactive [34]. This idea appears to be empirically born out in meerkats (*Suricata suricatta*), where dominant females who are reproductively inactive are less likely to be aggressive to subordinate females who attempt to breed [143]. Changes in reproductive value and effort might interact in important ways to shape age-based changes in social behaviour. For instance, declining residual reproductive value with age can also select for increased investment in reproduction [144] (e.g. terminal investment [145]). As a result, age-related increases in reproductive effort may lead to corresponding reductions in time and energy allocated to social effort, which run counter to the expected changes in social behaviour if one were to consider age-related declines in reproductive value alone [34]. Therefore, it is important to consider changes in both reproductive value and reproductive effort when considering the age-related drivers of social behaviour, which will require studies in systems with well-characterized lifetime social environments and patterns of life history (box 1).

### (c) Explanations for social ageing resulting from positive effects of age and demographic changes

Social ageing might also occur for reasons unrelated to senescence or secondary adaptations to senescence. There are also benefits that come with age, such as the increases in information, skill and experience that accumulate over an individual's lifetime, as well as shifts in the availability of kin, which might facilitate advantageous changes in social behaviour. Here, we discuss how age-based changes in social behaviour might be brought about by: (i) enhanced social experience and skills, and (ii) demographic effects and shifting kinship dynamics. As with all the previous sections, empirical evidence linking these explanations to social ageing is often limited, and our predictions for how social behaviour is expected to change with age are informed by theory whenever possible.

#### (i) Enhanced experience and skill with age

The longer an individual lives, the greater the number of opportunities they have to accrue important skills, experience and knowledge. In monkeys, whales and elephants, older individuals act as repositories of social and ecological information [32,33,146,147], making them valuable social partners. For example, older individuals can enhance group food location [32,146], navigation [148], and responses to climatological changes [149] and predatory threats [33]. Some of these benefits are thought to be due to transfer of knowledge from older to younger group members. If so, then older individuals should remain highly integrated in social groups late into life and make attractive social partners to other group members [150–152]. A lifetime of accumulated social experiences may also make older individuals more socially adept and better able to navigate social dilemmas, resolve conflict, and avoid exposure to negative stimuli. This might mean that older individuals are less often targets of aggression [8,15,89,150] and might make aged individuals less likely to engage in prosocial behaviour [8,11,150] as their need for social support wanes.

#### (ii) Demographic effects and shifting kinship dynamics across the lifespan

Age-specific changes in demographic schedules (i.e. in birth rates, death rates, and dispersal rates) and developmental traits cause the force of natural selection to change with age [34], which should influence age-based changes in social behaviour by altering who is available for interaction, including similarity to others in traits or states (their level of homophily), as well as familiarity and relatedness of group members. It has been shown, for instance, that similarity in age [13,151,153] and shared experience [154] are important drivers of the strength of social relationships. As an individual ages, they are likely to lose individuals with whom it has grown up and shared time, leading to the loss of important social connections. In this way, within-individual declines in sociality with age might occur as a result of population-level demographic changes. Local demography can also affect how average relatedness to potential social partners changes with age, which might result in advantageous within-individual shifts in social behaviour because individuals may gain indirect fitness by supporting relatives [155]. Theoretical models have demonstrated that changing patterns of relatedness can favour different patterns of helping and harming behaviour with age [156], which, in some specific contexts, is suggested to play an important role in selecting for late-life helping behaviour, such as the cessation of females' reproduction in favour of promoting their offspring's reproductive success [42,157]. However, in addition to affecting decisions ‘to breed’ or ‘not to breed’ [156], changes in local relatedness might also affect the rates and distribution of affiliative or agonistic interactions. Interspecies comparative work has demonstrated that when intragroup relatedness is high, affiliative behaviours tend to be undirected and widely distributed, with little social differentiation [158,159], while when intragroup relatedness is low, higher rates of aggression and more differentiated social relationships are common [158,160]. Interactions might also be expected to be more symmetrical where levels of kinship with potential partners (and therefore potential for inclusive fitness benefits) are low [158,161].

Kinship dynamics predict different patterns of age-related social behaviour depending on patterns of mating and individual dispersal. When mating occurs within social groups, mean relatedness to the group remains relatively constant for members that do not disperse at sexual maturity as relatives die or emigrate and are replaced by newly born relatives or immigrants [155,156]. As a result, we should expect that levels of social affiliation and social differentiation should be fairly stable with age for philopatric individuals. For dispersing individuals, mean relatedness to group members is expected to be low when they first join a group and to increase with age as they start to contribute offspring [155,156]. Dispersed individuals might therefore be expected to experience and engage in less aggression as they age and have larger and less differentiated social networks. In cases where dispersal is sex-biased, as is typically the case in mammals, with males dispersing more and farther than females [162], this is likely to produce different patterns of social ageing between the sexes [163]. Kinship dynamics can, however, still occur even when there is limited dispersal as a result of mating outside the social group [155,156]. When neither sex disperses but mating is non-local, individuals are born into a group without their father, but as they age and reproduce their sons will remain in the group, increasing relatedness to local males [155,156] and producing age-based changes in social behaviour.

## 3. Challenges in disentangling drivers of social ageing in mammals

To date, most studies of social ageing have focused on describing how patterns of behaviour vary as individuals age. This work provides the basis from which we can draw useful insights about how age can structure animal societies and how this can affect diverse ecological processes like disease transmission [164] and information acquisition [165]. However, similar patterns of social ageing can emerge from very different processes (figure 1) with different potential consequences for life history and senescence, thus illustrating the need to better understand the causes of social ageing.

By clearly laying out the potential drivers of social ageing in this review, we hope to facilitate future research on the causes of late-life changes in sociality. An important step in facilitating such future work is to not only summarize the potential drivers, but also highlight that these drivers are non-mutually exclusive. The seven explanations we have laid out in this review may occur at different levels of biological explanation (e.g. mechanisms §§2a(i–iii) and §2c(i) can be classed as proximate explanations, while §§2b(i,ii) and §2c(ii) can be classed, in some cases, as ultimate explanations) and should be expected to operate simultaneously and potentially synergistically. Establishing the relative contribution of these different explanations might prove particularly challenging as multiple mechanisms at the same level of explanation (i.e. proximate) can drive the same phenotypic outcome (figure 1), meaning researchers must be cautious in attributing social ageing to any one cause by looking at age-based changes in sociality alone. For instance, declines in the number of social partners with age (figure 1) might be the result of different patterns of biological senescence, including physical, energetic or cognitive ageing. Additionally, changes in social behaviour with age might simultaneously be the result of proximate mechanisms and ultimate drivers. For example, the physiological limitations and constraints that animals experience as they age might promote the need for adaptive allocation of resources and facilitate increased selectivity in social behaviour and partner choice. Some research has made strides in distinguishing the role of different explanations by demonstrating that older individuals tend to focus on preferred social partners, pointing towards greater selectivity [8,9,11,12] or showing that age-based differences in sociality may be driven by changes in spatial behaviour [16] or accompanied by declines in locomotor activities [8,35,56], which might suggest that physical or energetic limitations are also playing a key role in the process of social ageing.

However, in many cases, we are unlikely to be able to quantify the relative contribution of these explanations by studying behavioural outcomes alone. Advancing our understanding of the causes of social ageing will therefore require studies in systems with well-characterized patterns of life history and demography and where physical, cognitive and physiological markers of ageing can be measured alongside changes in social behaviour. In box 1, we illustrate the variety of integrative approaches that may be necessary not only to quantify the relative contribution of these different explanations but also to disentangle causation, as social ageing is likely to be a driver of biological senescence as well as a consequence of bodily decline. For instance, many studies support the idea that social status and social integration can play a causative role in disease and health outcomes. Reduced social integration and chronic social adversity enhance susceptibility to disease, illness and injury [166–168] and increase other markers of ageing and mortality risk [169–171]. Experimental manipulations and longitudinal studies that allow changes in social behaviour to be tracked across the lifespans of known individuals will be particularly helpful in determining whether within-individual age-related declines in physical or cognitive abilities precede or follow changes in sociality. Furthermore, distinguishing between age-related patterns that result from within-individual changes versus population-level processes (such as selective disappearance [38,39]) is an important distinction that future studies will need to make to advance our understanding of the ‘hows’ and ‘whys’ of social ageing. For instance, if less social individuals are more likely to die because of poorer access to resources, then an apparent age-related change in sociality might appear at the population level without necessitating any within-individual change. Longitudinal studies that follow known individuals through their lifetime will provide a key tool for differentiating these explanations and offering insight into when and how social ageing might evolve (box 1). Other biological explanations not mentioned here but that may be important, such as phylogenetic inertia, are also highlighted in box 1.

Social integration plays an important role in shaping access to resources [1] and thereby affects survival and reproductive success [2]. As a result, when social behaviour is age-dependent, it has the potential to modulate how fitness prospects change with age. Understanding the underlying causes of late-life changes in social behaviour is therefore important as social ageing may have the potential to either delay or accelerate other patterns of senescence [23,26]. For instance, we might expect that social ageing resulting from declines in bodily systems will lead to loss of important social relationships, resulting in negative fitness consequences in later life and therefore further exacerbating the rate of senescence. For example, initial senescent declines leading to social ageing may be further amplified in a positive feedback loop, whereby loss of social capital leads to loss of access to resources, facilitating a decline in physiological state, reducing reproductive output and increasing mortality risk [172]. Meanwhile, social ageing that results from positive effects of age or adaptive allocation of resources might lead to positive fitness outcomes and therefore might help to dampen senescence effects. For instance, the maintenance of social relationships into later life or adaptive narrowing of social networks might enhance access to resources, improving physical condition and increasing the reproductive success or survival of individuals in old age [172]. If this pattern of social ageing was widespread enough in the population, it could increase the force of selection and lead to the evolution of delayed senescence [26,173,174]. It should be noted, however, that these expectations are theoretical and that actual fitness costs or benefits associated with age-based changes in social behaviour have yet to be established and remain an important area for future research. Furthermore, recent research has suggested that there may be biological constraints on the extent to which ageing in humans and other mammals can be slowed [175]. Nevertheless, assessing both the causes of social ageing and the associated consequences for fitness will be critical both for integrating changes in sociality into our understanding of the natural ageing process and for providing evolutionary ecologists a deeper understanding of the causes of among-individual variation in patterns of ageing [176].

Social ageing is clearly a complex process that will require an integrative and multidisciplinary approach. While we have focused on group-living mammals owing to their similarity in social behaviour and phylogenetic proximity to humans, many social insects, fish and birds are likely to be informative in uncovering the diversity of patterns of social ageing in the wild as well as the underlying drivers of those patterns. Such study systems might also offer important insight into ageing interventions (e.g. [177]) or present new possibilities for disentangling causal relationships between social, physical, cognitive and physiological ageing [172,178–180]. Here, we have laid out several clear explanations for social ageing based on expected physical, cognitive, experiential, demographic and reproductive changes that occur across an individual's lifespan. We hope that researchers across disciplines will be able to use this review as a guide as they continue to work toward a deeper understanding of the physiological, ecological and evolutionary drivers of social ageing and in doing so facilitate a clearer picture of the role that social behaviour plays in the ageing process.

## Data Availability

No datasets were generated or analysed during the current study.
